# Exploring the Unmet and Met Needs of Homebound Older Adults: A Scoping Review

**DOI:** 10.1093/geroni/igaf122.2827

**Published:** 2025-12-31

**Authors:** Peace Kumapayi, Joseph Ogbuniro, Ravjyot Ughra, Benita Ugwu, Michael Kalu

**Affiliations:** York University, Toronto, Ontario, Canada; University of Nigeria Nsukka, Nsukka, Enugu, Nigeria; York University, Toronto, Ontario, Canada; University of Nigeria Nsukka, Nsukka, Enugu, Nigeria; York University, Toronto, Ontario, Canada

## Abstract

Homebound older adults, defined as individuals (65+) who remain indoors for extended periods without venturing outside experience significant challenges that negatively impact their physical activity, psychological health, and overall quality of life (1,2). Researchers have examined both the unmet and met needs of HB older adults, highlighting the necessity for a synthesis of this evidence. Therefore, this scoping review aims to synthesize existing evidence on the cognitive, environmental, financial, personal, physical, psychological, and social met and unmet needs of HB older adults.

**Method:**

Using Arksey and O’Malley’s (2005) framework, we searched across eight databases and supplemented by hand-searching reference lists of included articles. Screening (title, abstract, full text) and data extraction were conducted in pairs using predefined criteria. We employed thematic analysis (in pairs) to identify the met and unmet needs of HB older adults.

**Findings:**

A total of 19 articles were included, of which eleven were quantitative studies, seven were qualitative, one was mixed-method studies​. More than half of the included articles (n = 12) were conducted in the USA, with the remaining from China, United Kingdom, Portugal, Greece and Canada. Among the included articles, seventeen articles reported unmet needs ranging from cognitive (mild cognitive impairment), personal (lower life satisfaction), physical (functional impairment), psychological (depression), financial (financial constraints), environmental (housing needs) and social needs (loneliness). Remaining articles (n = 2) reported met needs using intervention such as Cognitive behavioural therapy (CBT) combined with Baduanjin qigong that reduced loneliness and Homebound elderly people psychotherapeutic interventions (HEPPI) that improved depression and episodic memory function.

